# FORUM: Ecological networks: the missing links in biomonitoring science

**DOI:** 10.1111/1365-2664.12300

**Published:** 2014-07-28

**Authors:** Clare Gray, Donald J. Baird, Simone Baumgartner, Ute Jacob, Gareth B. Jenkins, Eoin J. O'Gorman, Xueke Lu, Athen Ma, Michael J. O. Pocock, Nele Schuwirth, Murray Thompson, Guy Woodward

**Affiliations:** ^1^ School of Biological and Chemical Sciences Queen Mary University of London London E1 4NS UK; ^2^ Department of Life Sciences Silwood Park Imperial College London Buckhurst Road Ascot Berkshire SL5 7PY UK; ^3^ Department of Biology Environment Canada @ Canadian Rivers Institute University of New Brunswick 10 Bailey Drive P.O. Box 4400 Fredericton NB E3B 5A3 Canada; ^4^ Eawag—Swiss Federal Institute of Aquatic Science and Technology 8600 Dübendorf Switzerland; ^5^ Institute for Hydrobiology and Fisheries Science University of Hamburg Grosse Elbstrasse 133 22767 Hamburg Germany; ^6^ School of Electronic Engineering and Computer Science Queen Mary University of London London E1 4NS UK; ^7^ Centre for Ecology & Hydrology Maclean Building Benson Lane Crowmarsh Gifford Wallingford Oxfordshire OX10 8BB UK

**Keywords:** anthropogenic stress, climate change, conservation, food web, global warming, mutualism, pollination

## Abstract

1. Monitoring anthropogenic impacts is essential for managing and conserving ecosystems, yet current biomonitoring approaches lack the tools required to deal with the effects of stressors on species and their interactions in complex natural systems.
2. Ecological networks (trophic or mutualistic) can offer new insights into ecosystem degradation, adding value to current taxonomically constrained schemes. We highlight some examples to show how new network approaches can be used to interpret ecological responses.
3. *Synthesis and applications*. Augmenting routine biomonitoring data with interaction data derived from the literature, complemented with ground‐truthed data from direct observations where feasible, allows us to begin to characterise large numbers of ecological networks across environmental gradients. This process can be accelerated by adopting emerging technologies and novel analytical approaches, enabling biomonitoring to move beyond simple pass/fail schemes and to address the many ecological responses that can only be understood from a network‐based perspective.

## Abstract 2

Augmenting routine biomonitoring data with interaction data derived from the literature, complemented with ground‐truthed data from direct observations where feasible, allows us to begin to characterise large numbers of ecological networks across environmental gradients. This process can be accelerated by adopting emerging technologies and novel analytical approaches, enabling biomonitoring to move beyond simple pass/fail schemes and to address the many ecological responses that can only be understood from a network‐based perspective.

## Introduction

Biomonitoring programmes were born in the wake of the Industrial Revolution to measure the effects of environmental stressors on the natural world. Yet despite advances since then they are still unable to diagnose many perturbations, due to the paucity of baseline data, as well as a generally poor understanding of the underlying ecological mechanisms (Friberg *et al*. 2011). Most current programmes monitor changes in biodiversity or, increasingly, aspects of ecosystem functioning. Changes are assessed against a baseline level relative to a reference or idealised level (e.g. targets for restoration or acceptable levels of a response variable for that place and time). While the focus has typically been on monitoring taxonomic composition, the complex networks of species interactions that modulate ecosystem responses to stress have been ignored (Tylianakis *et al*. 2010; Friberg *et al*. 2011). A classic example of food web interactions determining alternative outcomes of both structural and functional responses to environmental stressors comes from shallow lakes. Here, catastrophic regime shifts are triggered by extreme nutrient concentrations, but in intermediate conditions trophic cascades in the food web can flip the ecosystem from one stable state to another, even in the absence of additional environmental change (Scheffer & Carpenter 2003). Ecological hystereses, whereby community recovery is modulated by the biota and not simply the reverse trajectory of the response to an impact (Scheffer & Carpenter 2003), highlight how the network of species interactions that underpin critical processes and services (such as pollination, clean water and fisheries) can influence both the internal dynamics of the system and its resilience to environmental change (e.g. Thompson *et al*. 2012).

Given the limitations of current biomonitoring, we argue that there is a need for new biomonitoring tools that (i) are grounded in the ecological processes underlying community responses to environmental change; (ii) can identify dominant stressors; (iii) can predict future responses; and (iv) can be applied across all aquatic and terrestrial ecosystem types. Here, we show how applying a network‐based approach can help to provide a new template for ecosystem biomonitoring.

## Theoretical foundations – ecological networks as biomonitoring tools

Traditional biomonitoring has focused on presence/absence or abundance of taxa (network ‘nodes’) across environmental gradients, while ignoring the network of pairwise interactions (‘links’) between them (Friberg *et al*. 2011). Ecosystem processes and the services they provide depend on interactions between individuals – which are frequently aggregated to species‐level (e.g. Tylianakis *et al*. 2010) or higher taxonomic or functional groupings. Interactions between these network nodes influence biodiversity and ecosystem functioning (Kremen 2005; Thompson *et al*. 2012) and a system's sensitivity to environmental change (Tylianakis, Tscharntke & Lewis 2007; see case study 1 below). Changes in network structure can provide clues to altered dynamics and ecosystem functioning, as demonstrated in a recent study that revealed habitat degradation reduced pollen transport for a focal plant species (Burkle, Marlin & Knight 2013). Current network approaches are still largely phenomenological (Tylianakis *et al*. 2010), but a more mechanistic, hypothesis‐led approach which considers relationships between network structure and ecosystem function is emerging (Heleno, Devoto & Pocock 2012), with recent examples including network responses to habitat restoration (Forup *et al*. 2008) and recovery from acidification (Layer *et al*. 2011).

Traditional biomonitoring is taxonomically grounded, limiting its ability to generalize beyond the characteristic biota of a given region or system. For instance, when assessing the ecological status of European rivers, huge effort has been devoted to harmonizing approaches and data across member states, forcing practitioners to resort to complex statistical intercalibration (see Birk *et al*. 2013). However, network approaches are not reliant on the taxonomy of the nodes *per se*, and so, in theory, can be used to compare emergent topologies of networks irrespective of biogeographical differences in species composition.

Keystone species, for instance, can be identified through a network approach (e.g. Jordán 2009), helping to focus monitoring efforts towards those that are ecologically most significant, since highly connected species often determine network stability and vulnerability to cascading secondary extinctions (e.g. Dunne, Williams & Martinez 2002). Similarly, a network approach can also help improve efficiency by identifying and tracking those species or interactions that are most sensitive to change; thus, keystone and indicator nodes could help provide novel early warning systems for detecting impending regime shifts or catastrophic ecosystem collapse (Aizen, Sabatino & Tylianakis 2012).

A network approach can help to reveal the complicated direct and indirect effects of stressors on an ecological community, beyond the simple loss or gain of species. For example, when freshwaters are acidified and specialist herbivores are excluded, generalist herbivore–detritivore species occupy their niche space, slowing their re‐establishment (e.g. Layer, Hildrew & Woodward 2013). Translocation experiments have shown that these acid tolerant consumers are generally not acidoiphilous *per se,* as they often perform just as well, if not better, in the absence of interactions with more acid sensitive species in the network. Empirical and modelling work has revealed that generalist acidified networks are more robust than their counterparts at higher pH, i.e. ecological inertia within the food web can modulate biological recovery as acidity ameliorates (Layer *et al*. 2010; Layer, Hildrew & Woodward 2013).

Network analysis has also revealed how another major environmental stressor – drought – leads to a top‐down erosion of stream food webs: large and rare species high in the web are especially sensitive and overall ecosystem functioning is compromised due to severely impaired biomass fluxes through the network (Ledger *et al*. 2013). The complex interconnected consequences of environmental stress for a particular system can thus only be fully understood from a network perspective, allowing *a priori* predictions to be made and appropriate management strategies to be developed. Ecotoxicology could also benefit from taking this more system‐based approach, as different pest control agents (insecticides, herbicides, fungicides) will affect different trophic levels and compartments in the food web, with ramifications that ripple far beyond the intended targets or other species with acute sensitivity to the poison: monitoring the network as a whole would help detect these potentially critical indirect and often unanticipated effects (e.g. Baird *et al*. 2001).

Environmental legislation increasingly requires both the structural and functional attributes of a particular community to be considered, but the latter are often still missing or inferred, despite increasing calls for them to be embedded in ecological assessments. Network approaches can help address this gap because many structural metrics are intimately linked to functioning (Thompson *et al*. 2012), although there is still an ongoing debate about which metrics are most ecologically informative and how sensitive they are to sampling intensity (e.g. Gibson *et al*. 2011). More sophisticated approaches (Blüthgen *et al*. 2007) and metrics for quantified networks are addressing these issues (e.g. Ulanowicz 2004; Tylianakis *et al*. 2010), but these have yet to be adopted explicitly in biomonitoring schemes.

Empirical studies have shown that some aspects of network structure can be sensitive to environmental change (e.g. Tylianakis, Tscharntke & Lewis 2007; case study 1, Layer *et al*. 2011; case study 2), whereas other metrics exhibit no clear response (e.g. Tylianakis, Tscharntke & Lewis 2007; Heleno, Devoto & Pocock 2012). Those properties that are more conserved (e.g. connectance) may be less sensitive, but when changes occur they could indicate imminent collapse or a regime shift as the system moves towards or crosses a tipping point. For instance, emergent network‐level properties (e.g. ascendancy, exergy; Ulanowicz, Jørgensen & Fath 2006) might be relatively resilient to perturbations up to a threshold, if redundancy among the nodes and links is sufficiently high that species turnover has little impact at these higher organizational levels. However, even if these high‐level properties are conserved, the structural rewiring of the web at the level of food chains or modules could have implications for other attributes of the system, such as the ability to retain particular taxa (Woodward *et al*. 2012). Such hierarchical responses could offer a range of new biomonitoring metrics related to the sensitivity and resilience of different organisational levels, from individual nodes to the whole network: network approaches will help us identify where those sensitivities lie, and to target management accordingly.

### Case‐Study 1: Tropical Host–Parasitoid Food Webs Response to Habitat Modification

**Figure 1:**
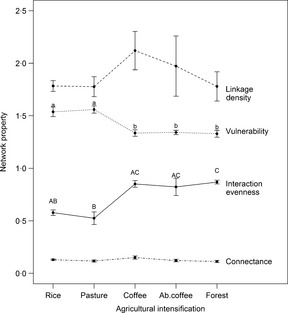
Effects of habitat modification on food web metrics (mean ± SEM) While some of the more traditional network metrics show no change across the gradient (connectance, linkage density) others, such as interaction evenness and node vulnerability, are sensitive to environmental degradation. Letters above individual means indicate significant differences among habitat types for that particular metric. Letters in common or no letters indicate no significant difference. Adapted from Tylianakis, Tscharntke & Lewis (2007).

Tylianakis, Tscharntke & Lewis (2007) constructed host parasitoid food webs along a habitat modification gradient, with both nodes and links quantified. They found dramatic changes in food web structure, which would not have been detected by traditional biomonitoring techniques since species richness did not vary across the habitat modification gradient. They found differences in network metrics such as interaction evenness and node vulnerability, but failed to detect changes in other metrics such as connectance and linkage density (Fig. 1) suggesting that the suitability of metrics for biomonitoring varies. These trends were lost when the analysis was repeated without information on the interaction strength, suggesting that quantitative information gives added value to ecological networks.

### Case Study 2: A Freshwater Food Web's Recovery from Acidification

**Figure 2:**
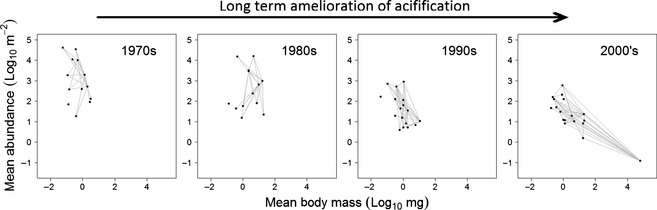
Broadstone stream food webs plotted as species abundance versus body mass data, with links between nodes representing trophic interactions. The abundance of invertebrates declines despite improving environmental conditions, as top‐down effects intensify. Redrawn from Layer *et al*. (2011).

The food web of Broadstone Stream is one of the most intensively studied ecological networks in the world (Layer *et al*. 2011). It is a relatively species‐poor food web but biomonitoring of this site has allowed recovery from anthropogenic acidification to be tracked over four decades, which culminated in the return of trout to this previously invertebrate‐dominated system. This amelioration of acidity, however, was not immediately followed by biological recovery: the community response did not simply show a straightforward reverse of the trajectory of the response to acidification, and invertebrate numbers actually declined as pH rose. These system‐level responses only made sense when viewed in the context of the food web: the declines in invertebrate numbers coupled with a succession of invasions of progressively larger predators, represented increasing top‐down effects and the resultant restructuring of the mass‐abundance scaling properties of the network (Fig. 2) even though the prey assemblage composition remained relatively constant. Traditional biomonitoring techniques could not explain this ecological response because they lacked the key ingredient: species interactions within the food web.

## Incorporating ecological networks into biomonitoring schemes

Although potentially useful, network‐based approaches must still overcome some significant challenges, particularly in terms of gathering data on interactions. In some cases, biomonitoring data are explicitly interaction‐based, e.g. monitoring pollinators by collecting individuals from flowers (as in Kremen, Ullman & Thorp 2011; Pocock, Evans & Memmott 2012) but, on the whole, direct monitoring of the interaction itself is currently too labour intensive to be practical in routine biomonitoring schemes (Hegland *et al*. 2010).

Where directly observing interaction data is impractical, one approach is to augment monitoring by inferring interactions based on prior knowledge and/or models. Such inferences are especially valuable where assemblages across trophic levels are routinely monitored, e.g. in aquatic systems (fish, macroinvertebrates and algae in freshwaters and whole fish assemblages in the sea). Interactions could be added from previously observed interactions, e.g. from data papers (e.g. Brose *et al*. 2005; Barnes 2008) and online resources, such as the Interaction Web Database (http://www.nceas.ucsb.edu/interactionweb/index.html) or the Database of Insects and their Food Plants (http://www.brc.ac.uk/dbif/). For instance, Mulder & Elser (2009) constructed a set of 22 food webs from biomonitoring data and published trophic interactions to show how chemical soil properties influence network structure and hence soil processes and services. Quantitative networks could be created from these known interactions based on simple rules (e.g. Pocock, Evans & Memmott 2012). Alternatively, interactions can be modelled directly from the occurrence data without reference to previously known interactions (e.g. size‐structured trophic models; Petchey *et al*. 2008; Woodward *et al*. 2010) or with other novel approaches (e.g. Bayesian belief networks, Milns, Beale & Smith 2010; text mining, Tamaddoni‐Nezhad *et al*. 2013). Where historic data exist (e.g. the UK Upland Waters Monitoring Network; Kernan *et al*. 2010) networks could even be inferred by hindcasting back through time.

Such inferred networks have potential limitations, however, as they ignore possible behavioural differences in species between systems, (i.e. preferential feeding depending on which resources are available) and unexpected or state‐specific changes in networks (e.g. those pre‐empting regime shifts) could go undetected. Data from inferred networks must therefore be tested and refined via iterative cycles of empirical observations, data quality checking and revalidation. Notwithstanding these caveats, the potential benefits are substantial, as the parameterization of networks using simple allometric scaling rules could ultimately allow interaction strengths or energy fluxes to be inferred and stability or productivity to be modelled dynamically (e.g. Berlow *et al*. 2009; Layer *et al*. 2010). This would provide a currently missing system‐level link between structure and (inferred) functioning. Inferring networks from the vast amounts of biomonitoring data already in existence brings the benefits of ecological network science into aspects of biomonitoring, while circumventing the huge effort required to construct each network anew from direct observation.

**Figure 3:**
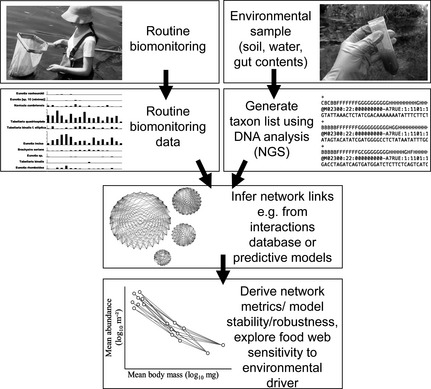
A conceptual diagram of how a networks based approach to biomonitoring can be incorporated into and work alongside traditional biomonitoring protocols through the use of Next‐Generation Sequencing technologies and a data base of ecological interactions.

An alternative approach to refining these existing approaches involves exploring better ways of constructing networks using new technologies. Next‐Generation Sequencing (NGS) and metasystematics offer huge potential for improving the taxonomic resolution and breadth of biomonitoring data, although challenges exist, e.g. for instance in obtaining accurate abundance estimates (e.g. Eiler *et al*. 2013). The diversity of microbes, macrobiota and even gut contents can now be described relatively easily using NGS (even to the level of resolving the internal microbiome network within consumers) (e.g. Shokralla *et al*. 2012). The steps involved in integrating these existing and emerging technologies for building ecological networks into an ecoinformatics‐based approach to biomonitoring can be represented as a flow chart (Fig. 3).

To support the use of ecological networks in biomonitoring, it is important to form *a priori* hypotheses rooted in ecological theory that will bring additional benefits (e.g. Heleno, Devoto & Pocock 2012) otherwise we will simply still be reporting patterns with no *a priori* predictive or explanatory power. A wealth of relevant network properties are routinely measured by ecologists (e.g. modularity, food chain length or connectance; see Thompson *et al*. 2012), which are supported by analysis and visualization packages such as r (R Core Team 2014), (e.g. cheddar; Hudson *et al*. 2013; or bipartite; Dormann, Gruber & Fruend 2008; igraph; Csardi & Nepusz 2006; or sna; Butts 2013). These online tools could be further developed to aid practitioners, since easily interpretable outputs could be generated, providing information about a given site's ecological status (for example the ‘window of vitality’; Ulanowicz 2002). Interdisciplinary collaboration will continue to allow the flow of ideas and novel metrics from other applications of network science, including biomedical research, social networks and information theory, into ecology (e.g. Ulanowicz 2004) to yield ever more sophisticated tools: the challenge now is to adopt and adapt these novel informatics approaches in a well‐informed way to add value to biomonitoring.

Just as the goals and aims of biomonitoring differ from site to site, the type of network monitored is likely to vary also, as the ecosystem services and functions they provide are prioritized differently from place to place. There is huge scope for further development in this area, e.g. in understanding the extent to which networks can withstand restructuring before the goods and services, which they provide become impaired (Tylianakis *et al*. 2010; Thompson *et al*. 2012). Some systems show clear signs in their network structure of impending regime shifts which have consequences for ecosystem functioning (e.g. Rawcliffe *et al*. 2010), whereas other networks experience significant network rearrangements without affecting some network metrics (Raffaelli & Friedlander 2012). Thus the interpretation of network data will depend on the type of system being monitored as well as the desired ecosystem goods and services.

## Conclusions

The ongoing global biodiversity crisis has received considerable attention, yet the associated losses of interactions that contribute to the degradation of ecological networks have often been overlooked. Since functional biodiversity is realised through interactions, environmental impacts on this aspect of biodiversity have profound implications for maintaining key ecosystem processes and services. We need to monitor the environment effectively and an ecological‐network approach, enhanced by new molecular and informatics techniques, offers a potentially fruitful avenue to develop a new generation of biomonitoring tools.

## Data accessibility

This article does not contain new data.
